# Micronutrient Intakes in 553 Dutch Elite and Sub-Elite Athletes: Prevalence of Low and High Intakes in Users and Non-Users of Nutritional Supplements

**DOI:** 10.3390/nu9020142

**Published:** 2017-02-15

**Authors:** Floris Wardenaar, Naomi Brinkmans, Ingrid Ceelen, Bo Van Rooij, Marco Mensink, Renger Witkamp, Jeanne De Vries

**Affiliations:** 1 HAN University of Applied Sciences, Sports and Exercise Studies, 6525 AJ Nijmegen, The Netherlands; Naomi.brinkmans@han.nl (N.B.); Ingrid.ceelen@han.nl (I.C.); Bo.v.rooij@gmail.com (B.V.R.); 2 Division of Human Nutrition, Wageningen University, 6700 EV Wageningen, The Netherlands; Marco.mensink@wur.nl (M.M.); Renger.witkamp@wur.nl (R.W.); Jeanne.devries@wur.nl (J.D.V.)

**Keywords:** dietary supplement, sport nutrition product, dietary intake, estimated average requirement, guidelines

## Abstract

This study investigated whether athletes meet micronutrient recommendations and whether the adequacy of their intake is related to the use of dietary supplements, sport nutrition products or a combination. Micronutrient intakes of 553 Dutch (sub-) elite athletes were assessed using web-based 24-h dietary recalls with accompanying nutritional supplement questionnaires. In the majority of both users and non-users of dietary supplements, vitamin D intake was below the estimated average requirement (AR) if supplements were not included in the analysis. Including dietary supplements improved vitamin D intake, but still a part of the athletes, both men and women, reported an intake below the AR. Non-users of dietary supplements were particularly at risk for low intakes of vitamins B1, B2, B3 and vitamins A, C and selenium. Mean iron intake was reported below the AR in a substantial group of women, both users and non-users. The use of sport nutrition products contributed only slightly to micronutrient intake. A small prevalence of athletes using dietary supplements showed intakes of some micronutrients above the Upper Level. In conclusion, both users and non-users of nutritional supplements reported inadequate intake of micronutrients. For most micronutrients, use of nutritional supplements does not completely compensate for intakes below AR. Athletes should consider making better food choices and the daily use of a low-dosed multivitamin supplement.

## 1. Introduction

Low micronutrient intake in athletes can result in deficiencies affecting health and performance, in particular when this occurs for longer periods of time [1]. Some studies report that many athletes do not meet micronutrient recommendations [2,3,4,5], whereas others conclude the opposite [1,6]. Shifts and variations in food patterns over time [7,8], increased availability of nutritional supplements [9], and changing viewpoints regarding requirements [10,11] merit regular monitoring of dietary intake by athletes. At the same time, rapid developments in assessment tools, such as web-based approaches, make it easier to gain insight in the intake of large groups of athletes [12,13].

No consensus exists on whether micronutrient requirements are different in athletes as compared to the general population [14,15]. In practice, athletes are nowadays often advised to meet the general recommended dietary reference intakes (DRI) for all micronutrients by consuming a diverse diet to ensure nutrient adequacy [16], paying special attention to optimal intake of iron, vitamin D and calcium, and of antioxidants [17]. There seems to be less attention on the intake of B-vitamins [17]. Theoretically, exercise could increase the need for this group of micronutrients [14]. However, if energy expenditure increases, food intake increases as well, which potentially could result in a higher vitamin B intake. Unfortunately this is not necessarily the case, in particular when athletes make poor dietary choices, resulting in a lower micronutrient intake than expected [14] because of a low micronutrient density of the diet [5,6,18]. 

Nutritional supplements are frequently used by athletes [19], although use can be irregular and varying over time [20]. Dietary supplements mainly comprise micronutrient supplements such as vitamins and minerals. These supplements may promote athletes’ general health through the prevention and treatment of nutrient deficiencies. On the other hand, sport nutrition products mainly contain macronutrients, such as carbohydrates and protein, but can also contain micronutrients. These sport nutrition products include, but are not limited to, sports drinks, recovery drinks, and sports bars. Finally, the category of ergogenic supplements, such as creatine and caffeine, may contain additional micronutrients although these products are also available as single bioactive substance products [21]. Therefore, nutritional supplements can be an important source of micronutrient intake.

Even though many athletes use multiple nutritional supplements at a time, this does not necessarily guarantee adequate habitual dietary intake of micronutrients at an individual level [7]. In addition, case reports have shown that some athletes consume very high doses of certain micronutrients exceeding the upper level (UL) also defined as tolerable upper intake level [11], possibly resulting in reduced health and performance in the long term [22]. Examples of frequently reported highly dosed micronutrient supplements used by individual athletes are antioxidants (i.e., vitamin C and E) [23], vitamin D, iron and magnesium [21]. Further, practical experience and anecdotal reports suggest a substantial use of high doses of vitamin B6 among athletes.

In the present study we aimed to evaluate the adequacy of micronutrient intake of Dutch elite and sub-elite athletes, using a web-based 24-h recall method with accompanying nutritional supplement questionnaires. In addition, we aimed to assess the effect of nutritional supplements use on micronutrient intake, by making a comparison between both users and non-users of dietary supplements, sport nutrition products or a combination of both. Ultimately this should lead to better identification of athlete groups at risk of inadequate micronutrient intake, either too low or too high.

## 2. Materials and Methods

### 2.1. Study Design

The study was performed between February 2012 and June 2015 as described by Wardenaar et al. [24] and others [25,26]. A total of 1800 athletes were informed about the study. A total of 759 elite and sub-elite athletes were interested in participating in this study and delivering three or four unannounced web-based, 24-h dietary recalls and questionnaires, preferably during a conditioning training phase. Written informed consent to participate was provided by all contacted, and for participants under the age of eighteen these documents were signed by their parents or guardian. No incentive was given for participation in the study. The survey was approved by the Medical Ethics Committee of Wageningen University.

### 2.2. Twenty-Four-Hour Recalls and Questionnaires

Dietary intake was estimated by combining information from web-based 24-h recalls and questionnaires as previously described for our validation study [13]. The 24-h recall method was based on the United States Department of Agriculture (USDA) five-step multiple-pass method [27,28], and was collected with “Compl-eat™”, a program built by the Division of Human Nutrition, Wageningen University. The web-based questionnaire, which was linked to the 24-h recall of a specific day, was collected with “Vitality portal”, a web-based questionnaire module built by the HAN University of Applied Sciences. By this questionnaire, characteristics (i.e., weight (kg), height (cm) and age in years), training load (total minutes of exercise per day) and nutritional supplement use were filled out by the participants or by a trained sports dietician during a face to face or telephonic interview. Nutritional supplements were defined as dietary supplements (i.e., micronutrients and essential fatty acids), sport nutrition products (i.e., carbohydrate and protein based products) or ergogenic supplements, for example creatine, b-alanine and other products with ergogenic claims [21]. For this study all products containing micronutrients, classified as dietary supplements (including ergogenic supplements) and sport nutrition products, were included in the analysis.

Two trained dieticians checked all web-based 24-h recalls and questionnaires for their completeness and any unusual portion sizes (i.e., incorrect amounts), and processed all notes made by the participants. If information on foods or nutritional supplements was missing, this was retrieved by contacting subjects. Adjustments for missing data and errors and dealing with relevant notes were done in a uniform way, using standard portion sizes and recipes according to a protocol [29]. Conversion of reported food consumption into energy and micronutrient intake was done using the Dutch food composition database of 2010 (Stichting NEVO, 2010), the Dutch database for dietary supplements (NES), and individual product ingredient declarations. To be able to correct for within-person variation, athletes had to deliver information for at least two complete days consisting of a complete 24-h recall and questionnaire. Results of the 24-h recall and questionnaires were combined to calculate: median (25–75 percentile) intake (g) of the most relevant basal nutrition food groups in relation to micronutrient intake (16 out of 22 provided by the Dutch food composition database), and total energy (MJ) and micronutrient intake (mg or µg) per gender and category.

### 2.3. Statistical Analyses

The statistical analysis included data of all athletes handing in at least two complete datasets using the statistical software program SPSS (version 23, IBM, Armonk, NY, USA). Prevalence of nutritional supplement use was reported as a percentage (%). Energy intake and micronutrient intake was reported as mean ± SD. Data was presented separately for men and women, for each of the four subgroups: non-users of nutritional supplements (‘non-users’), users of dietary supplements (DS), users of sport nutrition products (SNP) and users of a combination of both (DS + SNP).

Intake data was checked for normal distribution, by visual inspection of histograms and based on skewness and kurtosis. The intake distribution was adjusted for day to day variation using the following formula: SD_corrected_ = SD_observed_ × √ r_ic_ where r_ic_ is the intra class correlation coefficient (ᵖ = ᵟ_s_^2^/ᵟ_s_^2^ + ᵟ_e_^2^). Precision of the mean estimate (D_t_) of basal nutrition intake was acceptable around 10% for most micronutrients, and mostly below 20% [30]. The ratio of calculated energy intake/Basal Metabolic Rate (BMR) using Schofield’s formula [31] may be referred to as the food intake level (FIL) value, and was used to evaluate possible underreporting by comparison to a previously defined lower limit Physical Activity Level (PAL) of 1.55 [13].

The software program PC-SIDE (version 1.0), developed by the Department of Statistics and Center for Agricultural and Rural Development (CARD) at Iowa State University, was used to estimate distribution of usual intake [32]. This distribution was compared with the estimated average requirement (AR) based on Nordic Nutrition Recommendations [11] using the cut-off approach [33]. As for magnesium, only the Adequate Intake (AI) was available [11]; 75% of the AI was used as an estimator of average requirement. The prevalence (%) of athletes with an actual micronutrient intake exceeding the upper level (UL) [11] was calculated within each subgroup for all individuals using nutritional supplements. 

Differences within and between subgroups for each gender were analysed using Wilcoxon-signed-rank-test, Kruskall-Wallis and Mann-Whitney-U-test using a Monte Carlo approach. Statistical significance was set at *p* ≤ 0.05, no additional corrections were done for type II errors.

## 3. Results

A substantial part of the 759 athletes (*n* = 206) provided incomplete dietary information, i.e., less than 2 complete days. As a consequence, a total of 553 athletes, both men (59%) and women (41%), were included in the final analysis of this study. All results described in this paragraph are reported in Table 1. They recorded an average of 2.83 days by 24-h recalls and accompanying nutritional supplement intake questionnaires per person. For men and women respectively, 157 and 83 participants were classified as endurance athletes, 138 and 104 participants as team sports athletes, and 32 and 39 participants as strength athletes as described in Wardenaar et al. [24]. Mean age of the men was 23.5 ± 11.5 years and of the women 22.0 ± 7.6 years, and mean exercise time was 93.5 ± 61.3 min per day. Reported energy intake was on average 11.7 ± 3.2 MJ for men and 9.1 ± 2.5 MJ for women. Mean food intake level (FIL) values, as an indicator of the quality of energy reporting, were slightly higher for men (mean ± SD: 1.55 ± 0.39) than for women (1.50 ± 0.42) and differed between non-users and users of nutritional supplements resulting in a somewhat lower FIL in those not using supplements. Non-users of DS and SNP were mainly younger athletes in comparison to the other categories and particular users of DS + SNP were the oldest and reported the highest exercise load.

Of the total group of athletes, 61.8% reported the use of one or more nutritional supplements; 65% in men, and 56% in women. The use of DS alone was slightly lower in men (20%) than women (24%), while the use of SNP alone was more frequently reported by men (24%) than women (17%), as was the combined use of DS + SNP (men: 21%, women: 15%).

### 3.1. Basal Diet Intake

Food group intake is shown in Table 2 showing that median intake of athletes in all categories shows a divers consumption pattern. The most consumed food groups contributing to micronutrient intake were: milk products, meat, vegetables, cereals and grains and potatoes, fruit and bread. The consumption of eggs, fish and vegetarian meat-replacement products was low. In most cases, vegetable and fruit consumption was below the minimum recommendations of 150 grams and 2 servings respectively.

For men, micronutrient intake from the basal diet, i.e., excluding nutritional supplements, was in most cases lower in non-users than in all subgroups of users (Table 3, *p* < 0.05). In women this difference was less pronounced except for the users of DS + SNP, who reported higher mean intakes than non-users, DS users and SNP users (*p* < 0.05).

### 3.2. Total Micronutrient Intake

When nutritional supplements were included, users of DS and DS + SNP reported a higher total intake of all micronutrients as compared to the basal diet for both men and women (*p* ≤ 0.05). For SNP users (both men and women) the same trend was seen, except for vitamin A, vitamin D, iron, copper, selenium and zinc, for which values did not differ with the basal diet after including SNP in the calculation (*p* > 0.05).

Differences in mean intake existed between subgroups for almost all nutrients. Intake for most micronutrients was higher in users of DS and DS + SNP than in non-users and users of SNP in both men and women (*p* ≤ 0.05). In men, users of SNP also reported higher intake of most nutrients than non-users (*p* ≤ 0.05), except for vitamin A and D. Women using SNP only reported higher intakes than non-users for vitamin B2, B3, B6, folate equivalents and selenium (*p* ≤ 0.05).

### 3.3. Adequacy of Micronutrient Intake of the Basal Diet

Almost all athletes (>85%), both men and women, were not able to meet the AR of vitamin D (Table 4). The basal diet was also low for vitamin A (20%–48%) and vitamin B1 (18%–40%) for both men and women. Also, the intake of vitamin B2 was below the AR in some of the athletes, mainly in non-users and users of SNP in men and women (with a prevalence of low intakes varying between 12% and 17%), except for men using DS + SNP, who showed only a prevalence of low intakes of 4%. In general, non-users had the highest prevalence of micronutrient intakes below the AR. They showed low intakes of folate equivalents and selenium in both men (15% and 11% respectively) and women (25% and 11% respectively) as well as low intakes of vitamin C in men (11%). In addition, the basal diet of women, but not of men, showed within all subgroups of users and non-users, a prevalence of iron intakes below the AR. However, the overall athletes’ basal diet was providing sufficient amounts of vitamin B3, vitamin B12, vitamin E, calcium, phosphorus, magnesium, copper and zinc.

### 3.4. Adequacy of Total Micronutrient Intake including Nutritional Supplements

If nutritional supplements were included in the analyses, vitamin D intake of DS and DS + SNP users improved. The contribution of dietary supplements reduced the prevalence of athletes below the AR of vitamin D, but still low intakes were seen in 43% and 28% of the men and 19% and 35% of the women for DS or DS + SNP, respectively. Additionally, the use of DS improved intakes of vitamin B1, B2, folate equivalents and vitamin A, and ensured that most intakes exceeded AR. When the use of DS was included in the analysis, iron intake improved in DS-users. Women using DS + SNP showed no prevalence of intakes below the AR for iron whereas 9% of women reporting only DS still showed an iron intake below AR. In general, no beneficial effect on micronutrient intake was seen because of using SNP, except in women using SNP; the prevalence of women meeting AR for vitamin B1 and vitamin A improved.

### 3.5. Micronutrient Intake Exceeding UL

If the defined upper level (UL) was available for a specific micronutrient, this was shown in Table 5. The high exceedance of the UL for phosphorus in men was the result of a high intake from the basal diet. The prevalence of athletes with a micronutrient intake above the UL based on the basal diet without supplements (non-users and users of nutritional supplements excluding supplements) was in all other cases negligible.

The micronutrient intake of male users of nutritional supplements exceeded the UL for vitamin B3, in 22% and 16% using DS or DS + SNP, respectively. Of the women, 17% and 34% of users of DS or DS + SNP respectively were reporting above the UL for vitamin B3. In addition, the prevalence of high intakes of vitamin B6 (exceeding the UL with 9% and 11% for DS or DS + SNP users) and vitamin A (exceeding the UL with 8% and 17%) in women is worth mentioning. All other instances of micronutrients exceeding the UL were ≤4% in both men and women.

## 4. Discussion

In this survey among 553 elite and sub-elite Dutch athletes, it was shown that the basal diet alone, despite a reasonable diversity of reported food groups, often did not provide enough micronutrients to ensure adequate intake levels. However, when DS were included in the analyses, we saw significantly higher micronutrient intakes in users compared to non-users. Our study also revealed that vitamin D intake was a problem in all athletes. In addition, non-users of dietary supplements were particularly at risk for low intakes of vitamin B1, B2 and vitamin A. They also reported a low intake of vitamin B3, vitamin C and selenium, but this was less pronounced. Iron intake was below the AR in some but not all female athletes, but this appeared to be compensated for when dietary supplements were taken into account. Micronutrient intake above the upper level (UL) was only observed in those using DS, especially in case of vitamin B3.

### 4.1. Micronutrient Intake

The majority of the athletes (62%) involved in our study was using one or more dietary supplements, sport nutrition products or a combination of both, which is in line with a recently published meta-analysis [34]. On the other hand, DS and (or) SNP were not consistently used ‘daily’ in the current study, and not by all athletes. Nevertheless, reported micronutrient intake was substantial higher in athletes using supplements compared to those not using these products, which is in line with previously published data for the general Dutch population [35].

Remarkably, when nutritional supplements were not taken into account in the analysis, the intake of most micronutrients was still higher in users than in non-users, as was reported in Table 1. This difference in intake from the basal diet between users and non-users of dietary supplements and sport nutrition products may be a result of the higher total energy intake reported by users, because micronutrient intake correlates directly to energy intake for most micronutrients [7]. 

The current study did not reveal problems regarding vitamins B6 and B12, vitamins C and E, most minerals and trace elements as suggested by other studies [1,5,6,36]. This may be the result of a relatively high dairy and combined intake of bread, cereals and grains, which is common in the Netherlands [8]. 

#### 4.1.1. Vitamin D, Iron and B Vitamins

In all subcategories, the intake of vitamin D via basal diet was below the AR of 7.5 µg. Basal vitamin D intake was lower than previously reported for other groups of athletes [3,4,5,7], mainly because of the low consumption of vitamin D-containing foods such as butter and/or margarine, and of fatty fish in our population. An adequate vitamin D status in a young healthy population is associated with improved bone health and muscle strength and function [11,37]. Regarding bone mineral density, although low vitamin D intakes were seen, the calcium intake was above the AR and exceptionally low energy intakes were not seen in the present study [17]. When the use of vitamin D containing dietary supplements was included in the analysis, a substantial improvement of vitamin D intake was seen. Unfortunately, we did not assess the vitamin D status itself in our population, which is mainly determined by seasonal exposure to sunlight and dietary intake. Therefore, we can only speculate on the effect of the dietary intake of vitamin D on blood status. It was previously shown in a large group of Dutch athletes that they could be at risk for developing a low 25(OH)D status, especially during winter months [38]. Taking this into account, all athletes should be advised to use a dietary supplement containing 2.5–5 μg vitamin D in order to meet at least the suggested nutrition recommendations.

Almost one third of the female athletes experienced difficulties meeting the AR for iron through their basal diet, which confirms earlier observations in athletes [5,6,7,36]. A low iron intake increases the risk for iron deficiency or anaemia in which the oxygen-binding part of haemoglobin plays an important role [11]. In women, iron losses are generally higher through menstrual blood loss [39]. On the other hand, female athletes can suffer from amenorrhoea as a result of the high volume of exercise which could (temporarily) reduce the need for iron in the diet [40]. Athletes can also suffer from exercise-induced haemolysis, due to mechanically induced damage to red blood cells, which results in a higher iron loss by the body [41]. A low dietary iron intake in combination with exercise-induced haemolysis does not necessarily lead to iron deficiency (anaemia), as for example was seen in gymnasts [41]. Anyway, the AR is suggested as the lower minimal level that should be met by all athletes. Athletes should be advised to select proper sources of iron, from both animal and plant-based sources, and the use of a multivitamin and mineral supplement by women could add to dietary iron intake in order to meet the recommended levels.

A substantial fraction of both non-users and users of only SNP reported an intake below the AR for vitamin B1 and B2 (thiamine and riboflavin, respectively) in both men and women, and for folate equivalents in women. Theoretically, exercise increases the need for vitamin B1 and B2, as a result of a decreased absorption and (or) an increased turnover related to tissue maintenance, repair and metabolism [14]. It is generally assumed that athletes with a poor thiamin and riboflavin status have a reduced ability to perform physical activity, especially performing maximal work [14]. Previously, vitamin B1 and B2 were not seen as a particular nutritional problem for Dutch athletes [7]. A comparable prevalence for low intakes of folate were reported in 20%–25% of Dutch women of the general population [8]. The observed energy intake in the present study was not exceptionally high in comparison to the Dutch general population. Possibly this resulted in lower B vitamin intake as confirmed by others [1,5,6,36], except for vitamin B2 [1,5,6,36]. To cover the requirements of specific B vitamins, athletes should be encouraged to frequently select vitamin B1 rich foods (for example lean meat, legumes that were almost not present in the diet of these Dutch athletes) and vitamin B2 rich foods (for example eggs, lean meat and specific dairy products).

#### 4.1.2. Anti-Oxidants

Exercise is associated with increased oxidative stress [42,43,44]. Notwithstanding the continuing debate on the relation between their oral intake and the anti-oxidant status of the body, athletes are currently advised not to use anti-oxidant supplements as they could negatively influence protein signaling and adaptation in relation to oxidative stress [42,43,44]. Although the bioactive anti-oxidative substances with the diet transcends the micronutrients included in this study, the basal dietary intake of vitamin C, vitamin E, selenium and zinc was in most cases above the AR in the present study. Only the basal intake of vitamin A, reported in this study as retinol activity equivalents (i.e., including b-carotene), was low in a substantial number of all athletes. Therefore, consuming a well-chosen diet, rich in a large variety of fruits and vegetables containing b-carotene and retinol containing products, should be considered to optimize the antioxidant capacity of the diet [17,45].

### 4.2. Micronutrients Exceeding UL

The prevalence of micronutrient intake exceeding the UL based on dietary supplement use was very low, i.e., a prevalence of 1%–4%, with the exception of users of dietary supplements containing nicotinic acid (vitamin B3), vitamin B6 and (or) vitamin A. High vitamin B intakes, as a result of the use of dietary supplements, could lead to side effects like flushing, and to more severe health problems (vitamin B3) [11] and neuropathy (vitamin B6) [46].

In the current study, eighteen athletes reported an intake of vitamin B6 above the generally accepted UL of 25 mg [47]. Therefore, athletes should be advised to check their dietary supplements and avoid a combination of (highly dosed) nutritional supplements. 

The UL is defined to prevent toxicity due to excessive micronutrient consumption over a long term. However, for example, high intake levels of vitamin C and E below the UL have been associated with attenuated skeletal muscle adaptations and protein signaling [43,48]. In the current study, no high doses of vitamin E were reported, but a substantial number of athletes reported the use of high-dosed vitamin C supplements (i.e., 1000 mg or higher). At present no consensus exists on the effect of high doses of vitamin C on redox signaling [23], thus athletes should preferably maintain an appropriate vitamin C status by selecting a large variety of fruits and vegetables.

### 4.3. Strengths and Limitations

The large elite athletic population and the unannounced 24-h recalls and questionnaires were a strong aspect of our study. Also, dietary reports included two week days and one weekend day. By using the method as described by Nusser et al. (1996), we removed the within-person variation and were able to compare the distribution of usual intake with the average requirement [32]. With the inclusion of three days for almost all participants, the method was suitable to sufficiently correct for within-person variation [49]. Based on low D_t_, we conclude that the effect of random errors for most nutrients was low, except for vitamin B1 in women not using nutritional supplements which makes it more difficult to draw conclusions for this value than for the others [30]. 

A limitation of this study is that we did not have the possibility to measure micronutrient concentrations in blood. On the other hand, good biochemical indicators of dietary intake or status are not available for all micronutrients, or they only reflect short-term micronutrient intake. There is only a limited number of biomarkers available to assess usual micronutrient intake (for example folic acid concentrations in erythrocytes reflecting intake of the past six weeks), especially when only single measurements are taken [29]. Also, concentration biomarkers in blood cannot be used to estimate absolute levels of intake. Thus, although self-reports of micronutrients have errors and inter-individual differences exist regarding individual needs, it can be assumed that the percentage of micronutrient intakes below the AR reflect the best estimate of the percentage of the study population with intakes that are below their individual requirements [32]. Nevertheless, we recommend that future research also takes into account micronutrient status because it provides objective information in addition to the self-reports.

All self-reporting methods are prone to misreporting and may not completely reflect true dietary intake [12,49]. Based on our previous validation study we know that our method, similarly to other self-reports, underestimates intake [13]. Therefore, we calculated the FIL as an estimator for possible energy under-reporting. The FIL is especially lower for the non-users of supplements in both men and women. As the non-users were reporting the lowest workload (exercise minutes per day) it is expected that they also report lower energy intakes. Although FIL values for non-users are substantially lower than those of users of nutritional supplements, this falls within an acceptable range [13]. If we assume that micronutrient intake was not biased by selective under-reporting, correcting for under-reporting would overall result in a higher intake per person and thus actual micronutrient intake would be somewhat higher and inadequacy lower than presented. 

Our study shows that there is still room for improvement of the athletes’ diet. With regards to the use of dietary supplements, several suggestions can be made for well-informed use. These suggestions are provided by Table 6.

## 5. Conclusions

In this large athletic population, a substantial part of non-users as well as users of nutritional supplements reported an inadequate intake of some micronutrients, in particular vitamin D, iron, vitamin B1, B2 and vitamin A. The additional use of dietary supplements in general improved intake levels, but this did not always completely compensate for intakes below the average requirement. Only a small proportion of athletes was at risk for chronic consumption of high doses of single micronutrients above the UL, with vitamin B3 as the most pronounced example. As there is room for improvement of micronutrient intake in the athletes’ diet, athletes should make better food choices resulting in a higher micronutrient intake and consider the option of using a daily (low-dosed) multivitamin supplement containing 50%–100% of the Recommended Intake (RI).

## Figures and Tables

**Table 1:** Mean ± SD of characteristics, duration of exercise and energy intake per day, and food intake level (FIL) for the total group and subgroups of athletes.

Characteristics	Men (*n* = 327)				Women (*n* = 226)			
	Non-Users	Users DS	Users SNP	Users DS and SNP	Non-Users	Users DS	Users SNP	Users DS and SNP
	A	B	C	E	F	G	H	I
Number ( *n*) and %	115 (35%)	67 (20%)	77(24%)	68 (21%)	99 (44%)	53 (24%)	39 (17%)	35 (15%)
*Endurance (n)*	*36*	*40*	*44*	*37*	*33*	*21*	*9*	*20*
*Team (n)*	*68*	*25*	*20*	*25*	*55*	*19*	*20*	*10*
*Strength (n)*	*11*	*2*	*13*	*6*	*11*	*13*	*10*	*5*
Height (cm)	177 ± 12.4	180 ± 10.4	183 ± 7.5 ^a^	184 ± 8.1 ^a,b^	173 ± 6.7	172 ± 9.9	171 ± 9.7	174 ± 8.6
Weight (kg)	67.7 ± 15.3	70.7 ± 13.3	75.3 ± 9.9 ^a,b^	78.4 ± 12.0 ^a,b^	64.8 ± 7.3	63.1 ± 9.9	64.6 ± 9.9	66.3 ± 8.8
BMI (kg^−1^·m^2^)	21.3 ± 2.9	21.6 ± 2.4	22.5 ± 2.0 ^a,b^	23.0 ± 2.3 ^a,b^	21.8 ± 1.9	21.3 ± 2.2	22.0 ± 2.2 ^g^	22.0 ± 2.3
Age (year)	21.1 ± 11.9	22.3 ± 10.6 ^a^	26.4 ± 12.4 ^a,b^	25.3 ± 9.7 ^a,b^	20.3 ± 5.7	22.5 ± 9.2	20.0 ± 4.4	28.1 ± 9.2 ^f,g,h^
Exercise time (min^−1^·day)	69 ± 59	75 ± 39 ^a^	96 ± 56 ^a^	96 ± 47 ^a,b^	95 ± 63	117 ± 78	117 ± 69 ^f^	131 ± 59 ^f^
Energy intake (MJ)	10.5 ± 2.4	11.7 ± 3.6 ^a^	12.5 ± 3.4 ^a^	12.9 ± 3.2 ^a,b^	8.7 ± 2.0	8.9 ± 3.1	9.4 ± 2.3	10.5 ± 2.6 ^f,g^
Food intake level (FIL)	1.44 ± 0.3	1.54 ± 0.4 ^a^	1.62 ± 0.4 ^a^	1.64 ± 0.4 ^a^	1.40 ± 0.3	1.47 ± 0.5	1.52 ± 0.4	1.73 ± 0.4 ^f,g,h^

Capital letters (A–I) are used as a label for subgroups for gender. Lower case letters displayed below a specific value indicate that this value is significantly larger than the corresponding column defined with a capital. Significance based on Mann-Whitney U test was set at *p*-value of 0.05, significant values of *p* ≤ 0.001 were displayed with a cursive lower case.

**Table 2:** Median (25–75 percentile) intake (g) of basal nutrition food groups per gender and category.

Food Groups	Men				Women			
	Non-Users	Users DS	Users SNP	DS + SNP	Non-Users	Only DS	Only SNP	DS + SNP
	(*n* = 115)	(*n* = 67)	(*n* = 77)	(*n* = 68)	(*n* = 99)	(*n* = 53)	(*n* = 39)	(*n* = 35)
Bread	210 (158–265)	210 (148–290)	226 (135–305)	202 (148–272)	153 (98–218)	120 (69–176)	142 (95–188)	129 (72–223)
Cereals and cereal products	73 (20–136)	93 (30–203)	121 (55–194)	137 (55–263)	90 (51–172)	83 (46–134)	112 (52–184)	117 (45–209)
Potatoes	54 (0–100)	75 (33–133)	67 (24–123)	50 (0–129)	30 (0–80)	38 (0–98)	30 (0–83)	33 (0–103)
Vegetables	82 (43–153)	148 (90–229)	137 (85–201)	122 (73–199)	117 (67–196)	171 (102–255)	147 (74–236)	243 (149–345)
Fruits	128 (43–230)	146 (64–301)	176 (77–286)	163 (53–293)	220 (114–309)	228 (144–317)	200 (127–287)	267 (171–383)
Cheese	20 (0–43)	21 (7–37)	16 (0–42)	23 (7–40)	18 (5–32)	13 (0–30)	13 (3–24)	14 (2–31)
Milk and milk products	311 (175–450)	292 (199–492)	388 (256–619)	309 (177–542)	280 (180–410)	361 (116–544)	364 (205–500)	283 (158–426)
Eggs	1 (0–25)	0 (0–33)	17 (0–34)	25 (0–67)	17 (0–33)	6 (0–33)	25 (0–47)	27 (0–50)
Fish	0 (0–0)	0 (0–42)	0 (0–49)	0 (0–53)	0 (0–17)	0 (0–40)	0 (0–8)	21 (0–52)
Meat, meat products and poultry	121 (84–163)	130 (72–171)	131 (68–188)	172 (107–216)	97 (59–134)	100 (60–129)	114 (99–153)	97 (40–156)
Soy products and vegetarian products	0 (0–0)	0 (0–0)	0 (0–0)	0 (0–0)	0 (0–0)	0 (0–0)	0 (0–0)	0 (0–33)
Fats, oils and savory sauces	40 (20–61)	47 (25–69)	34 (18–66)	45 (28–64)	33 (19–58)	28 (19–43)	27 (15–48)	22 (13–45)
Pastry and biscuits	29 (8–56)	34 (13–67)	35 (15–66)	27 (6–58)	28 (16–48)	22 (8–44)	25 (7–57)	37 (10–94)
Sugar, sweets and sweet sauces	30 (15–52)	34 (18–52)	30 (11–56)	30 (11–53)	17 (9–37)	17 (8–36)	18 (8–43)	26 (14–46)
Nuts, seeds and snacks	10 (0–27)	15 (0–40)	13 (0–34)	21 (0–47)	10 (0–25)	9 (0–25)	7 (0–19)	9 (0–27)
Beverages *	1160 (783–1731)	1333 (949–2316)	1483 (946–2268)	1709 (1160–2484)	1133 (749–1516)	1500 (946–2277)	1307 (935–1770)	1750 (1291–2266)

Of a total 23 food groups, the following were not included in this table: miscellaneous foods, savory bread spreads, mixed dishes, herbs and spices, legumes, clinical formulas and soups with a median value of 0. * Alcoholic and non-alcoholic beverages.

**Table 3:** Mean ± SD intake of micronutrients in users and non-users of nutritional supplements (mg or µg) for men and women.

Micro-Nutrients	Men							Women						
	Non-User	Users DS		Users SNP		Users DS and SNP		Non-User	Only DS		Only SNP		DS + SNP	
	(*n* = 115)	(*n* = 67)		(*n* = 77)		(*n* = 68)		(*n* = 99)	(*n* = 53)		(*n* = 39)		(*n* = 35)	
		Excluding	Including	Excluding	Including	Excluding	Including		Excluding	Including	Excluding	Including	Excluding	Including
	A	B	C	D	E	F	G	H	I	J	K	L	M	N
Vitamin B1 (mg)	1.3 ± 0.3	1.5 ± 0.5	6.6 ± 11.8 ^a,b,e,g^	1.6 ± 0.5 ^a^	1.7 ± 0.5 ^a,d^	1.5 ± 0.3 ^a^	5.8 ± 7.1 ^a,e,f^	1.1 ± 1.4	1.2 ± 0.4	11.3 ± 12.4 ^h,i,l,n^	1.2 ± 0.3	1.3 ± 0.3 ^k^	1.2 ± 0.2	10.8 ± 7.7 ^h,l,m^
Vitamin B2 (mg)	1.8 ± 0.4	1.9 ± 0.5	7.7 ± 10.8 ^a,b,e^	2.3 ± 0.7 ^a,b^	2.3 ± 0.7 ^a,d^	2.0 ± 0.6	7.6 ± 14.8 ^a,e,f^	1.6 ± 1.5	1.7 ± 0.4	14.0 ± 19.7 ^h,i,l^	1.8 ± 0.5	1.9 ± 0.3 ^h,k^	1.7 ± 0.4	11.8 ± 7.4 ^h,l,m^
Vitamin B3 (mg)	22 ± 6	26 ± 6 ^a^	46 ± 20 ^a,b,e^	28 ± 7 ^a^	29 ± 7 ^a,d^	28 ± 6 ^a^	51 ± 30 ^a,e,f^	18 ± 2	21 ± 3	43 ± 23 ^h,i,l^	22 ± 5	24 ± 4 ^h,k^	21 ± 4 ^h^	54 ± 24 ^h,l,m^
Vitamin B6 (mg)	2.1 ± 0.6	2.3 ± 0.6	6.8 ± 4.4 ^a,b,e^	2.5 ± 0.9	3.3 ± 2.5 ^a,d^	2.3 ± 0.4 ^a^	6.8 ± 6.5 ^a,e,f^	1.8 ± 1.5	2.2 ± 1.0 ^h^	11.4 ± 13.1 ^h,i,l^	2.3 ± 0.5 ^h^	2.5 ± 0.2 ^h,k^	2.1 ± 0.4 ^h^	12.4 ± 8.3 ^h,l,m^
Vitamin B11 (mg)	257 ± 50	311 ± 62 ^a^	568 ± 190 ^a,b,e^	339 ± 83 ^a^	353 ± 91 ^a,d^	333 ± 78 ^a,b^	627 ± 197 ^a,e,f^	252 ± 61	295 ± 89 ^h^	758 ± 354 ^h,i,l^	289 ± 85	295 ± 84 ^h,k^	345 ± 100 ^h^	678 ± 211 ^h,l,m^
Vitamin B12 (mg)	4.2 ± 0.8	5.6 ± 0.9 ^a^	13.4 ± 13.6 ^a,b,e^	6.2 ± 1.9	7.4 ± 4.3 ^a,d^	6.6 ± 1.7 ^a^	12.3 ± 14.7 ^a,e,f^	4.2 ± 1.8	4.2 ± 0.4	113 ± 730 ^h,i,l^	4.3 ± 0.5	5.4 ± 1.4 ^k^	4.6 ± 1.0	23.8 ± 16.7 ^h,l,m^
Vitamin A: RAE (µg)	723 ± 120	773 ± 208	1189 ± 266 ^a,b,e^	812 ± 164	814 ± 164	752 ± 177	1284 ± 778 ^a,e,f^	654 ± 65	650 ± 228	1194 ± 618 ^h,i,l^	677 ± 42	686 ± 62	922 ± 415 ^h,i,k^	1639 ± 637 ^h,l,m^
Vitamin C (mg)	113 ± 49	145 ± 52 ^a^	285 ± 170 ^a,b,e^	138 ± 37 ^a,f^	153 ± 80 ^a,d^	119 ± 32 ^b^	345 ± 183 ^a,e,f^	116 ± 29	124 ± 45	319 ± 187 ^h,i,l^	126 ± 39	134 ± 38 ^k^	163 ± 65 ^h,i^	396 ± 249 ^h,l,m^
Vitamin D (µg)	3.2 ± 0.6	3.9 ± 1.0 ^a^	10.0 ± 9.1 ^a,b,e^	4.3 ± 1.4	4.3 ± 1.4	5.3 ± 0.8 ^a,d^	10.7 ± 8.1 ^a,e,f^	3.3 ± 0.6	3.1 ± 1.3	20.6 ± 10.9 ^h,i,l,n^	3.3 ± 0.7	3.4 ± 0.6	3.8 ± 1.7	7.7 ± 4,l.7 ^h,l,m^
Vitamin E (mg)	13 ± 3	16 ± 5 ^a^	30 ± 18 ^a,b,e^	15 ± 4	16 ± 4 ^a,d^	18 ± 5 a	32 ± 14 ^a,e,f^	12 ± 3	14 ± 6	45 ± 33 ^h,i,l^	12 ± 2	13 ± 3 ^k^	16 ± 2 ^h,k^	35 ± 17 ^h,l,m^
Calcium (mg)	970 ± 181	1066 ± 234	1159 ± 245 ^a,b,e^	1251 ± 253 ^a,b^	1296 ± 243 ^a,d^	1163 ± 231 ^a^	1377 ± 362 ^a,c,f^	920 ± 190	1037 ± 159 ^h^	1135 ± 211 ^h,i,l^	971 ± 299	989 ± 284 ^k^	1086 ± 227 ^h^	1212 ± 261 ^h,l,m^
Phosphorus (mg)	1598 ± 209	1836 ± 390 ^a^	1853 ± 382 ^a,b^	2063 ± 374 ^a^	2074 ± 363 ^a,d^	2102 ± 385 ^a,b^	2172 ± 397 ^a,c,f^	1466 ± 190	1626 ± 356 ^h^	1636 ± 356 ^h,i^	1606 ± 355	1607 ± 354	1724 ± 339 ^h^	1764 ± 329 ^h,m^
Magnesium (mg)	363 ± 60	449 ± 114 ^a^	502 ± 142 ^a,b^	478 ± 98 ^a^	493 ± 88 ^a,d^	487 ± 123 ^a^	560 ± 117 ^a,c,e,f^	351 ± 68	415 ± 195 ^h^	475 ± 250 ^h,i,l^	382 ± 90	390 ± 88 ^k^	465 ± 132 ^h,k^	524 ± 143 ^h,l,m^
Iron (mg)	12 ± 2	15 ± 3 ^a^	19 ± 5 ^a,b,e^	16 ± 3 ^a^	16 ± 3 ^a^	16 ± 4 ^a^	21 ± 5 ^a,e,f^	12 ± 2	12 ± 4	18 ± 5 ^h,i,l,d^	13 ± 3	13 ± 3	15 ± 3 ^h,i^	25 ± 22 ^h,l,m^
Copper (µg)	1.3 ± 0.2	1.6 ± 0.4 ^a^	2.0 ± 0.5 ^a,b,e^	1.6 ± 0.3 ^a^	1.7 ± 0.3 ^a^	1.7 ± 0.4 ^a^	2.2 ± 0.5 ^a,e,f^	1.3 ± 0.2	1.5 ± 0.9	2.1 ± 0.9 ^h,i,l^	1.3 ± 0.3	1.3 ± 0.3	1.6 ± 0.4 ^h,i,k^	2.3 ± 0.8 ^h,l,m^
Selenium (µg)	48 ± 8	58 ± 17 ^a^	77 ± 28 ^a,b^	65 ± 17 ^a^	65 ± 18 ^a^	80 ± 24 ^a,b,d^	100 ± 31 ^a,c,e,f^	46 ± 9	60 ± 46 ^h^	95 ± 49 ^h,i,l^	51 ± 7 ^h^	51 ± 8 ^h^	62 ± 13 ^h^	80 ± 24 ^h,l,m^
Zinc (mg)	11 ± 1	13 ± 2 ^a^	17 ± 4 ^a,b,e^	14 ± 2 ^a^	14 ± 2 ^a^	15 ± 3 ^a,b^	20 ± 5 ^a,e,f^	10 ± 2	11 ± 3	18 ± 6 ^h,i,l^	11 ± 2	11 ± 1	12 ± 2 ^h,i^	19 ± 5 ^h,l,m^

All nutrient intake values are based on actual intake. Capital letters (A–I) are used as labels for subgroups for gender. Lower case letters displayed below a specific value indicate that this value is significantly larger than the corresponding column defined with a capital. Significance was set at *p*-value of 0.05. Differences were tested within categories (excluding/including nutritional supplements) based on the Wilcoxon-signed rank test using the Monte Carlo approach with *p* ≤ 0.05. Differences were tested between categories for nutrient intake excluding nutritional supplements and including nutritional supplements based on the Mann-Whitney U test using the Monte Carlo approach with *p* ≤ 0.05.

**Table 4:** Adequacy of micronutrient intake (%) in men and women presented for users and non-users of nutritional supplements.

Micro-Nutrients	Men							Micro-Nutrients	Women						
	Non-User (*n* = 115)	Users DS (*n* = 67)		Users SNP (*n* = 77)		Users DS+SNP (*n* = 68)			Non-user (*n* = 99)	Users DS (*n* = 53)		Users SNP (*n* = 39)		Users DS+SNP (*n* = 35)	
		Basal Diet	Including DS	Basal Diet	Including SNP	Basal Diet	Including DS + SNP			Basal Diet	Including DS	Basal Diet	Including SNP	Basal diet	Including DS + SNP
Vit B1 (1.2 mg)	36	18	5	21	17	26	6	Vit B1 0.9 mg	40	21	1	18	9	10	0
Vit B2 1.4 mg	16	17	2	12	11	15	5	Vit B2 1.1 mg	13	11	3	17	12	4	0
Vit B3 15 mg	11	5	0	4	4	7	1	Vit B3 12 mg	0	3	3	2	0	1	0
Vit B6 1.3 mg	7	5	1	7	5	3	1	Vit B6 1.0 mg	2	3	2	0	0	0	0
Folate eq. * 200 µg	15	4	0	2	2	4	1	Folate eq. * 200 µg	25	14	3	21	18	5	0
Vit B12 1.4 mg	0	0	0	0	0	1	0	Vit B12 1.4 mg	0	1	1	0	0	3	0
Vit C 60 mg	11	3	0	3	3	4	0	Vit C 50 mg	3	2	1	1	1	1	0
RAE 600 ug	20	34	6	28	28	27	10	RAE 500 ug	23	48	14	31	10	15	3
Vit D 7.5 µg	100	95	43	86	86	87	28	Vit D 7.5 µg	100	98	19	100	100	90	35
Vit E 6.0 mg	1	0	0	5	3	1	0	Vit E 5.0 µg	1	1	0	0	0	0	0
Ca 500 mg	1	1	0	1	0	0	0	Ca 500 mg	3	1	0	8	5	0	0
P 450 mg	0	1	0	0	0	0	0	P 450 mg	0	0	0	0	0	0	0
Fe 7.0 mg	0	1	0	0	0	0	0	Fe 10.0 mg	38	30	9	25	18	10	1
Mg 263 mg	4	5	2	2	1	1	1	Mg 210 mg	3	2	1	3	2	0	0
Cu 0.7 mg	0	1	0	0	0	0	0	Cu 0.7 mg	1	1	0	2	1	0	0
Se 35 µg	11	8	2	3	3	2	0	Se 30 µg	11	7	2	0	0	1	2
Zn 6.0 µg	0	0	0	0	0	0	0	Zn 5.0 µg	0	0	0	0	0	0	0

All proportion values are based on the cut-off approach (% < AR) after intake distribution estimation of habitual intake based on the ISU approach [32]. Proportion for magnesium is estimated based on 75% of the available Adequate Intake (AI) for men and women. * Folate eq. = Folate equivalents.

**Table 5:** Prevalence (%) of athletes exceeding Upper Level (UL) of selected micronutrients based on mean intake for men and women split by non-users and users of nutritional supplements.

Micronutrients	UL	Men							Women						
		Non-Users	Users DS		Users SNP		Users DS + SNP		Non-Users	Users DS		Users SNP		Users DS + SNP	
			Basal Diet	Including DS	Basal Diet	Including SNP	Basal Diet	Including DS + SNP		Basal Diet	Including DS	Basal Diet	Including SNP	Basal Diet	Including DS + SNP
Vitamin B3 (mg)	35 mg	-	-	1	-	22	-	16	-	-	0	-	17	-	34
Vitamin B6 (mg)	25 mg	0	0	1	0	4	0	4	0	0	0	0	9	0	11
Vitamin A: RAE (µg)	3000 RE	0	0	0	0	0	0	4	0	0	0	0	8	0	17
Vitamin D (µg)	100 µg	0	0	0	0	0	0	0	0	0	0	0	4	0	0
Vitamin E (mg)	300 mg	0	0	0	0	0	0	0	0	0	0	0	0	0	0
Calcium (mg)	2500 mg	0	0	0	0	1	0	4	0	0	0	0	0	0	0
Phosphorus (mg)	3000 mg	0	8	8	3	3	9	9	0	0	0	2	2	0	0
Magnesium (mg)	350 mg	-	-	0	-	0	-	3	-	-	0	-	2	-	3
Iron (mg)	60 mg	0	0	0	0	0	0	0	0	0	0	0	0	0	3
Copper (µg)	5.0 µg	0	0	0	0	1	0	0	0	0	0	2	2	0	6
Selenium (µg)	300 µg	0	0	0	0	0	1	1	0	0	0	2	2	0	0

All nutrient intake values are based on actual intake. UL = Upper Level, based on Nordic Nutrition Recommendations. [11] Prevalence is given as a percentage of respondents with average micronutrient intake exceeding UL. * The used UL for Vit B3 and Mg is only based on a maximal supplementation value of 35 mg and 350 mg, therefore dietary intake is not taken into account for this analysis. - indicates that no exceedance of UL can be calculated based on basal dietary intake excluding nutritional supplements.

**Table 6:** Suggestions for supplement users: dos and don’ts.

**What Athletes Should Do:**
Identification of low micronutrient status as a part of dietary assessment, for example by measuring 25-OH-D levels (vitamin D status) and hemoglobin and ferritin levels (iron status) in the blood, possibly extended with using the most appropriate evaluation measurements for other vitamins. Proper selection of dietary supplements that are complementary to the daily diet. Daily use of 5–25 mcg vitamin D and a low-dosed multivitamin (50%–100% of the Recommended Daily Intake (RI)) to guarantee meeting the recommendations. Make use of available quality assurance programs for dietary supplements, sports nutrition products and ergogenic aids (i.e., informed choice for the US, informed sport for UK, Koelner Liste for Germany or Nederlands Zekerheidssysteem Voedingssupplementen Topsport (NZVT) for the Netherlands). All athletes making use of nutritional supplements (i.e., dietary supplements, sport nutrition products and ergogenic aids) should make a table summarizing the totals of all supplements and nutrients consumed per day and cross check this with a sport dietitian/nutritionist.
**What Athletes Should Not Do:**
Irregular use or not well-considered use of nutritional supplements in disagreement with scientific consensus. Supplement high doses of so-called antioxidant nutrients such as vitamin A (or b-carotene), vitamin C, vitamin E and selenium, but instead consume a well-balanced diet containing antioxidant-rich foods. Exceed defined Upper Limits for single nutrients by using extremely high-dosed dietary supplements or by combining multiple products. Make use of any products that are subject to adulteration and contamination.
